# Ionic Gelation for Nano-Delivery of Sulforaphane in Animal Feed: A Conceptual Review of Stability, Efficacy, and Translation Potential

**DOI:** 10.3390/biology15131045

**Published:** 2026-06-30

**Authors:** Kevaun Altamon George Wilson, Mengke Zhang, Yiming Shen, Mukesh Kumar, Sandreika Osheika Laird, Salwa Eman, Jun Xu, Haibing Li, Mengzhi Wang, Xiaodong Guo

**Affiliations:** 1 College of Animal Science and Technology, Yangzhou University, Yangzhou 225009, China; kevaun.wilson@case.edu.jm (K.A.G.W.); 13218975259@163.com (Y.S.); dh24021@stu.yzu.edu.cn (M.K.); mh24126@stu.yzu.edu.cn (S.E.); 2 College of Food Science and Light Industry, Nanjing Tech University, Nanjing 211816, China; zhangmengke0725@163.com; 3 Center for Biological Control, College of Agriculture and Food Sciences, Florida A&M University, Tallahassee, FL 32307, USA; sandreika1.laird@famu.edu; 4 Institute of Animal Husbandry and Veterinary Medicine, Jiangxi Academy of Agricultural Sciences, Nanchang 330200, China; 5 Yangzhou Kerunde Machinery Co., Ltd., Yangzhou 211400, China; lhb@kerunde.com; 6 Joint International Research Laboratory of Agriculture & Agri-Product Safety of MOE, Yangzhou University, Yangzhou 225009, China

**Keywords:** nanotechnology, sulforaphane, bioavailability, ionic gelation, encapsulation, feed additives

## Abstract

Sulforaphane (SFN), a bioactive compound sourced from cruciferous vegetables, offers significant antioxidant and anti-inflammatory benefits yet its stability in animal feed is a challenge. Nanotechnology-based encapsulation, specifically ionic gelation, has demonstrated efficacy in improving the stability and bioavailability of SFN. This review examines the application of natural polymers such as chitosan and alginate in ionic gelation for the encapsulation of SFN. It also discusses how these polymers can prevent SFN from degrading while traversing the digestive tract. Encapsulation strategies for SFN have been associated with improved bioavailability, with absorption reported in experimental modules, to modulate immune-related and oxidative-stress pathways. However, challenges persist in identifying optimal methods for encapsulating various species, including enhancing encapsulation effectiveness, particle size, and controlled release mechanisms. Additionally, regulatory concerns regarding the safety and environmental impacts of nanoparticles in feed must be addressed. Future research should focus on improving encapsulation techniques and ensuring the safe application of SFN-loaded nanocarriers in livestock feed.

## Simple Summary

Sulforaphane (SFN) is a bioactive phytochemical with antioxidant and anti-inflammatory properties, but its volatile nature in animal feed limits its efficacy. This review examines how sulforaphane can be securely transported through the digestive system by microscopic natural particles composed of chitosan and alginate, shielding it until the animal takes it. Animals may receive more nutrition, more robust immune responses, and improved general health by employing this strategy. The review highlights the translational potential of these techniques for poultry, swine, and ruminants. The majority of current research focuses on human and rodent models and thus this scarcity defines the research gap our work addresses. Important factors are covered, such as controlled release, encapsulation effectiveness, particle size optimization, and regulatory compliance. This conceptual framework identifies potential to improve animal nutrition and lays the groundwork for upcoming practical studies on SFN delivery in animal feed.

## 1. Introduction

**Figure 1:**
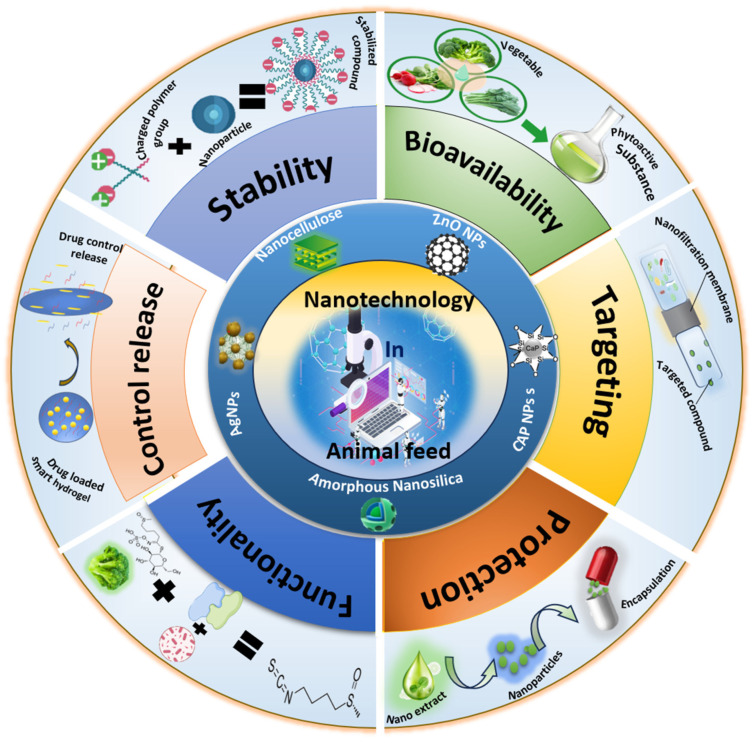
A systematic review of nanotechnology applications in livestock feed and nutrition.

Sustainable livestock production has become a critical focus in light of the increasing global demand for animal-derived products and the need to reduce the environmental footprint of animal farming. Efficient use of feed resources is central to achieving sustainability in this sector. Nanotechnology-based feed strategies have been increasingly explored to improve the stability, bioavailability, controlled release, targeting, protection, and functionality of bioactive compounds in livestock nutrition (Figure 1). One promising bioactive compound, sulforaphane (SFN), is recognized for its antioxidant, anti-inflammatory, and anticancer properties, making it a potential addition to livestock feed [1,2]. However, despite its therapeutic potential, SFN faces a significant challenge due to its physicochemical instability and low bioavailability when used in feed. The compound’s inherent instability, especially under the digestive conditions in livestock, limits its bioavailability and prevents it from exerting its full beneficial effects [3,4]. This issue is compounded by the complexity of the digestive system in livestock, which makes it difficult for bioactive compounds like SFN to maintain efficacy after ingestion [5].

Nanoencapsulation is widely recognized as a promising strategy to improve the bioavailability of bioactive compounds. By encapsulating SFN within nanocarriers, researchers aim to enhance its stability and control its release over time, addressing its instability issues [6,7]. While several studies have investigated nanoencapsulation as a solution to improve SFN’s bioavailability, many of these efforts fail to consider an engineering perspective tailored to the complexities of feed systems [8]. Moreover, there is often a lack of understanding about how the encapsulation process interacts with the diverse components of feed, which could affect the bioactive compound’s stability and functionality [9]. While previous reviews have summarized the bioactivity and encapsulation of sulforaphane in human and rodent models [10,11,12], none have systematically addressed the design principles, material selection, and in-feed performance of nanoencapsulation for livestock. Evidence from pigs, broilers, and ruminants demonstrates that nano-encapsulated SFN can improve growth, liver function, and resilience, but species-specific challenges such as ruminal degradation remain. Therefore, a clear gap remains in understanding how encapsulation strategies, such as ionic gelation, can be optimized for practical SFN delivery in livestock feed systems [10]. These materials are not only biocompatible but also provide inherent tunability, making ionic gelation an ideal platform for the encapsulation of sensitive bioactive compounds like SFN [11]. Previous studies have shown that ionic gelation can improve the stability of bioactive compounds in feed systems, but the current body of knowledge remains fragmented. While there is evidence that ionic gelation can improve SFN stability, a systematic framework linking the design principles of ionic gelation to its stability and efficacy outcomes in feed has yet to be fully established [12,13]. Furthermore, the specific interactions between gelation materials and SFN within the complex feed matrix require further investigation to optimize delivery systems [14].

The central thesis of this review is that ionic gelation represents a rational and designable platform for the controlled delivery of SFN in livestock feed. This review will present a detailed exploration of the design principles underlying ionic gelation, followed by its application to engineer SFN stability. This paper will further address the efficacy of these systems, demonstrating how ionic gelation-based formulations can enhance the bioavailability and therapeutic potential of SFN in feed systems. Finally, this work consolidates examine practical strategies for integrating ionic gelation-based SFN delivery systems into commercial feed formulations, bridging the gap between laboratory findings and real-world applications in the livestock industry [15,16]. Through this conceptual critical review, we propose a structured framework for evaluating ionic gelation as a potential SFN delivery strategy in livestock feed. This framework integrates evidence on SFN instability, polymer selection, encapsulation performance, controlled release, species-specific digestive challenges, and regulatory considerations. It is intended as a design roadmap for future experimental validation rather than as an experimentally validated protocol.

## 2. Ionic Gelation as an Engineering Framework for Nano-Delivery

### 2.1. Principle of Ionic Gelation

**Figure 2:**
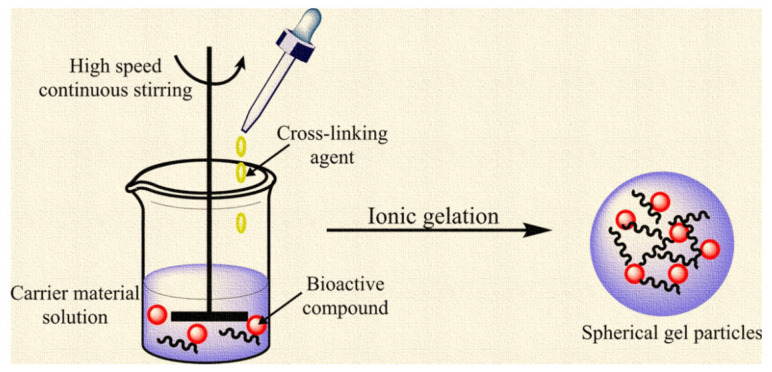
Diagram illustrates the process of ionic gelation. Adapted from [23]. Licensed under CC BY 4.0. Image colors were enhanced. Bioactive chemicals are incorporated into a carrier solution. The combination is subsequently poured into an ionic solution, causing droplets to rapidly cross-link into spherical gel particles [24].

Ionic gelation is a prevalent technique in nanotechnology for the fabrication of hydrogels and nanoparticles by connecting polymer chains, forming a network structure. The fundamental concept of ionic gelation is employing multivalent ions to cross-link biopolymer chains via electrostatic interactions. This creates a three-dimensional network architecture. The ionic strength, pH, and quantity of cross-linkers can significantly alter this process. This affords substantial control over the characteristics of the resulting gel [17,18]. Ionic gelation operates by forming physical cross-links via ionic interactions rather than covalent connections. Chitosan, a cationic polymer, can interact with multivalent anions such as tripolyphosphate (TPP) to form nanoparticles by ionic crosslinking. The degree of gelation and the physicochemical properties of the resulting particles, including size, charge, and stability, are influenced by variables such as polymer concentration, cross-linker ratio, pH, and ionic strength [19]. A recent study demonstrated that homogenization and precise pH regulation can reduce particle size, enhancing their stability and dispersibility. This is particularly significant for environmental and biological applications [20]. Ionic gelation facilitates the creation of dual-crosslinked (Figure 2) or modified nanoparticles that exhibit enhanced performance. A study demonstrated that covalently and ionically crosslinked chitosan nanoparticles, utilizing genipin and TPP, exhibited improved stability and novel antibacterial characteristics via quorum sensing suppression, thereby expanding the applicability of this approach [21]. Additionally, scalable technologies such as static mixing have been developed to facilitate ionic gelation on a large scale, enabling the consistent and repeatable production of chitosan nanoparticles [22]. These principles form the basis for designing SFN-loaded carriers that maximize stability and controlled release in animal feed applications.

### 2.2. Application for Sulforaphane Encapsulation

Ionic gelation is an effective method for encapsulating sulforaphane, employing a gentle, aqueous technique that avoids high temperatures and chemical solvents that may degrade this delicate molecule. Sulforaphane is recognized for its instability, rapidly degrading when subjected to heat, oxygen, and variations in pH. Ionic gelation encapsulation forms protective polymeric matrices that preserve bioactivity even in mild conditions [12]. This approach has been demonstrated to significantly prolong the shelf life of sulforaphane and ensure its safety throughout storage and transit through the digestive system. Biopolymers such as sodium alginate, chitosan, and carrageenan are frequently employed to produce ionic gels. Alginate, a negatively charged polymer derived from brown seaweed, forms gels upon interaction with divalent cations such as calcium. Chitosan is a cationic polymer derived from crab shells. Upon interaction with alginate or other anionic polymers, it generates robust, biocompatible gels. These polymers are environmentally friendly, decompose organically, and are frequently utilized in food and pharmaceuticals. The majority of studies on sulforaphane encapsulation have examined several techniques, including spray drying and coacervation. Nonetheless, the principles of ionic gelation remain significant and are considered effective methods for stabilizing sulforaphane [25].

### 2.3. Advantages of Ionic Gelation

Ionic gelation enhances the stability of encapsulated compounds such as sulforaphane [12,26]. The polymeric coating formed during gelation safeguards sulforaphane from environmental challenges such as heat, oxidation, and pH fluctuations, which are recognized for rapidly degrading its bioactive properties [27]. Ionic gelation facilitates controlled release, which is crucial for maximizing the therapeutic benefits of sulforaphane. For example, encapsulated sulforaphane can be released in the intestines, where its bioavailability is enhanced and absorption is facilitated [28]. Furthermore, encapsulation via ionic gelation enhances resistance to environmental degradation. This entails safeguarding sulforaphane from degradation caused by heat or oxygen during production or storage. Studies consistently show that encapsulated sulforaphane has superior absorption and extended pharmacological effects compared to its unencapsulated counterpart [29]. The biodegradability of alginate–chitosan matrices enhances their safety for application in food and pharmaceutical health advantages of sulforaphane in the animal sector.

## 3. Engineering Stability: Encapsulation Design and Protective Performance

### 3.1. Nano Extraction of Sulforaphane

**Figure 3:**
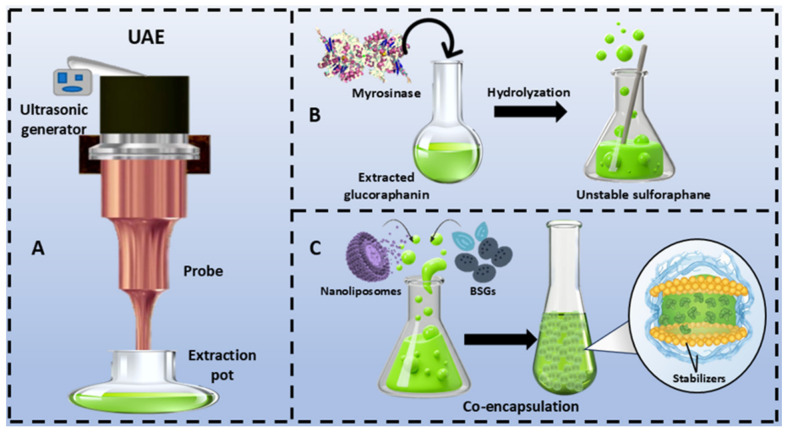
Overview of the process for enhancing the bioavailability of sulforaphane from broccoli. (**A**). UAE (ultrasound-assisted extraction) of broccoli to extract glucoraphanin. Modified from [39] licensed under CC BY 4.0. (**B**). The enzyme-mediated transformation of glucoraphanin into sulforaphane [6]. (**C**). Enhancing and stabilizing sulforaphane through co-encapsulation with nanoliposomes and basil seed gum to improve its bioavailability [40]. The colors are used for visual differentiation of the sequential process steps and key components: the blue background separates the extraction, hydrolysis, and co-encapsulation stages; green represents broccoli-derived glucoraphanin/sulforaphane-containing solutions; black arrows indicate process direction; and dashed borders define individual schematic panels. The colors are illustrative only and do not represent quantitative differences.

Ultrasonic-assisted extraction (UAE) is a prevalent method for extracting sulforaphane from broccoli seed or sprout extracts. This technique shown in Figure 3A enhances both the yield and purity of the active ingredient [7]. The efficacy of UAE is attributed to sonic cavitation, which accelerates mass transfer and facilitates the enzymatic degradation of glucoraphanin, the precursor of sulforaphane, as shown in Figure 3B. This indicates that the UAE can significantly enhance and expedite the extraction process [30]. Additional investigations demonstrated that ultrasonic treatment enhanced sulforaphane extraction efficiency by 2.7 times compared to conventional shaking methods, hence validating the method’s efficacy [31].

These findings have also been corroborated by large-scale industrial applications. Patents delineate ultrasonication combined with enzymatic hydrolysis and solvent extraction as an economical, scalable, and selective method for producing high-purity sulforaphane [32,33]. The combination of UAE with techniques like microwave pretreatment or optimized solvent ratios further boosts the sulforaphane yield, making it a reliable method for both laboratory and commercial extraction processes [34]. The UAE is a rapid, efficient, and reproducible method for obtaining sulforaphane from broccoli-derived sources.

### 3.2. Enzymatic Hydrolysis of Glucoraphanin

Myrosinase can hydrolyze glucoraphanin enzymatically to release active sulforaphane. The enzymatic hydrolysis of glucoraphanin by myrosinase is a crucial process in the natural production of sulforaphane, a bioactive compound recognized for its potent antioxidant and anticancer properties. Myrosinase, present in broccoli and other cruciferous vegetables, accelerates this reaction when the cells are disrupted via during cutting or mastication. This converts glucoraphanin, which is typically inactive, into sulforaphane. Research has demonstrated that activated myrosinase significantly improves the bioavailability of sulforaphane. A study indicated that glucoraphanin, when provided with endogenous plant myrosinase, had 3 to 4 times more bioavailability compared to its administration without myrosinase [6]. Biotechnological advancements have facilitated the synthesis of recombinant myrosinase enzymes, such as Rmyr from *Rahnella inusitata*, capable of converting glucoraphanin to sulforaphane with an efficiency of 92% in under 10 min [35]. Genetically engineered yeast cells capable of producing myrosinase demonstrated around 100% effectiveness in converting glucoraphanin from broccoli seeds into sulforaphane, even after many uses [36]. Moreover, alternative sources of myrosinase, such as Chinese flowering cabbage, have been examined, revealing effective conversion of glucoraphanin to sulforaphane under optimal pH and temperature conditions [37]. These data collectively confirm that enzymatic hydrolysis by myrosinase is a natural, scalable, and efficient method for generating sulforaphane from glucoraphanin.

### 3.3. Enrichment of Sulforaphane

Co-encapsulation of sulforaphane (SFN) with stabilizers such as lecithin and plant-derived gums has emerged as an effective method to enhance its stability and bioactivity. Sulforaphane is recognized for its anticancer and antioxidant properties yet it is highly unstable due to its sensitivity to variations in pH, temperature, and enzymatic activity. Ref. [28] conducted a study demonstrating that encapsulating sulforaphane-enriched broccoli sprout extract in lecithin-based nanoliposomes, further stabilized with basil seed gum (BSG), significantly enhanced its stability and controlled release (Figure 3C). The optimal formulation exhibited a diminutive particle size (39.6 nm), a substantial zeta potential (−71.16 mV), and an exceptional encapsulation efficiency (97.96%). This co-encapsulation facilitated the release of the medication in the intestines, potentially enhancing its bioavailability [28].

García-Saldaña et al. effectively microencapsulated sulforaphane using a combination of gelatin, gum Arabic, and pectin through a method known as complex coacervation. These biopolymer complexes prevented the degradation of SFN and maintained its functional integrity, achieving encapsulation rates of 80% [25]. Zambrano et al. found that employing gum Arabic to encapsulate sulforaphane in oil-in-water emulsions enhanced its stability at elevated temperatures by sixfold and reduced its susceptibility to degradation under heat [38]. The findings indicate that incorporating lecithin and plant-derived gums, such as basil seed gum, gum Arabic, or pectin in co-encapsulation techniques improves formulation stability and control release characteristics.

### 3.4. Encapsulation of Sulforaphane via Ionic Gelation

**Figure 4:**
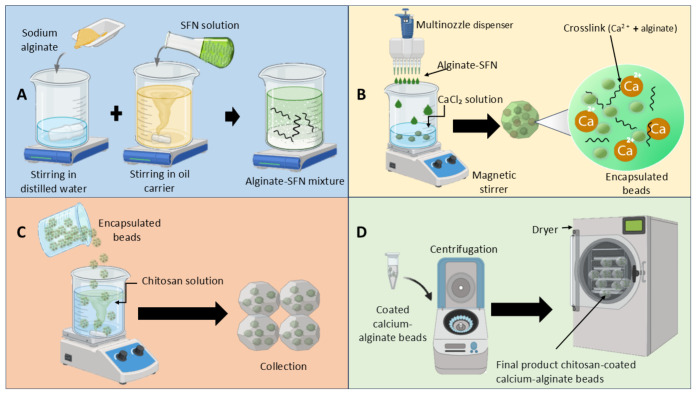
(**A**) Sodium alginate is blended with SFN dissolved together with vegetable oil, yielding a homogenized alginate–SFN mixture [44]. (**B**) Dispensing and gelation process is done by drop size the calcium–alginate mixture in a bath of calcium chloride (CaCl_2_) [45]. (**C**) Secondary coating using chitosan forms a polyelectrolytes complex that improves mechanical strength, reduces permeability and offers a better protection to the encapsulated agent [46]. (**D**) Post processing, the coated capsules are collected, centrifuged to remove excess supernatant and put to dry under suitable conditions. SFN, a polyphenol, has been encapsulated using ionic gelation to improve its bio accessibility and stability [47]. The colored panels represent the sequential preparation steps: blue, mixture preparation; yellow, gelation and crosslinking; orange, chitosan coating; and green, centrifugation/drying. The “+” symbol indicates mixing, black arrows indicate process direction, green particles represent SFN-loaded alginate beads, and “Ca” denotes calcium ions involved in alginate crosslinking.

Chitosan and sodium tripolyphosphate (TPP) are two well studied ionic gelation techniques that effectively encapsulate bioactive compounds such as sulforaphane. Chitosan, a naturally occurring polysaccharide with a positive charge, interacts with TPP, which possesses a negative charge, to form stable nanoparticles or microcapsules in aqueous solutions at room temperature. This method is effective for thermolabile compounds such as sulforaphane, which may decompose when subjected to high temperatures or aggressive chemicals. The chitosan–TPP system offers numerous advantages, including biocompatibility, biodegradability, and mucoadhesiveness, rendering it ideal for oral or mucosal delivery systems [41]. Studies demonstrate that ionic gelation can produce nanoparticles ranging from 100 to 500 nm, with surface charges (zeta potentials) between +20 and +40 mV, indicating considerable colloidal stability [42]. Encapsulation efficiencies over 60% have been achieved, even for compounds with limited water solubility or high degradation susceptibility [43]. While individual synthesis of ionic gelation encapsulation have been reported separately such as alginate–SFN homogenization [44] (Figure 4A), calcium chloride gelation [45] (Figure 4B), and chitosan coating [46] (Figure 4C), complete, integrated synthesis of the SFN-loaded alginate–chitosan encapsulation system as depicted in Figure 4A–D is not directly supported by any single published study. Therefore, the proposed sequential synthesis from homogenization to post-processing (Figure 4) represents a conceptual integration presented in this review, integrating individually reported steps into a unified SFN-loaded alginate–chitosan encapsulation strategy. However, this proposed workflow should be regarded as a design framework for future validation rather than an experimentally validated protocol [47].

However, there is a lack of precise studies that capitalize on encapsulating SFN using IG for animal feed. Although ionic gelation shows promise for protecting SFN from degradation and improving its delivery, studies on its use in animal feed remain limited. More in vivo livestock studies are needed to confirm its effectiveness under practical feeding conditions [47,48]. Table 1 summarizes representative encapsulation strategies for sulforaphane (SFN) and related isothiocyanates, highlighting both direct SFN studies and analogues that inform potential animal-feed applications. Taken together, these findings should be interpreted as formulation-level evidence supporting SFN encapsulation systems rather than direct proof of improved livestock performance. Most current data are derived from in vitro systems, pharmaceutical models, or limited animal studies, and therefore do not yet confirm predictable outcomes under practical feeding conditions.

**Figure 5:**
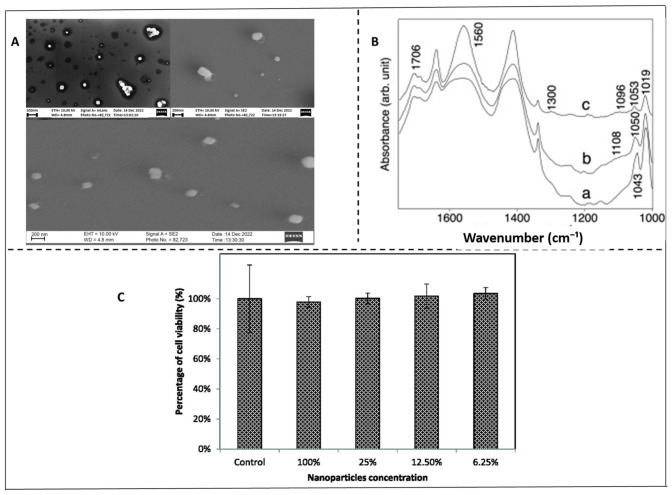
(**A**) Scanning electron microscopy (SEM) characterization of chitosan nanoparticles (CNPs) after synthesis via homogenization and subsequent purification by filtration. Reproduced from [20]. Licensed under CC BY 4.0. (**B**) Fourier-transform infrared (FT-IR) spectra of (a) pristine chitosan, (b) chitosan–silica nanohybrids, and (c) chitosan–silica/CpG ODN nanohybrids. Reproduced from [58] with permission from Elsevier. Copyright (2013). (**C**) Assessment of chitosan nanoparticle cytotoxicity in RAW-Blue murine macrophages using the CCK-8 assay. Reproduced from [60]. Licensed under CC BY 4.0.

Chitosan nanoparticles with scanning electron microscopy (SEM) as a secondary size characterization method in addition to DLS. SEM images were taken for CNPs synthesized with a homogenizer and purified with syringe filtration, as this procedure proved to be effective in formulating <100 nm CNPs with low polydispersity. The CNPs displayed a circular morphology, similar to the literature, and were split into two size populations, with average sizes of 75 ± 8 nm and 33 ± 11 nm [20] (Figure 5A). Figure 5B FT-IR analysis evidently demonstrates that chitosan can be successfully functionalized with silica to form stable nanohybrids, and further complexed with Cytosine-phosphodiester-guanine oligodeoxynucleotides (CpG ODNs) through interactions involving its amide groups [57]. These spectral changes confirm the ability of chitosan nanoparticles to act as effective nanocarriers, preserving their chemical functionality while integrating bioactive molecules [20,58]. Thus, chitosan nanoparticles should be highly recognized not only as a biocompatible scaffold but also as a flexible platform for delivering immunostimulatory oligonucleotides in vaccine or immunotherapy applications. Additionally, ionic chitosan gels prepared with tripolyphosphate were reported as biocompatible with low toxicity in mouse fibroblast-like NIH-3T3 cells during 24 h and 72 h incubation [59]. In line with these results, the current study assessed the cytotoxicity of chitosan nanoparticles to RAW-Blue cells utilizing the CCK-8 assay, as illustrated in Figure 5C. The cells were kept in a nanoparticle solution for four hours at different concentrations. The nanoparticles did not significantly affect cell viability; instead, cell viability increased in direct correlation with nanoparticle dilution [60].

### 3.5. Conceptual Dosage Framework of Sulforaphane via Ionic Gelation Encapsulation

With reported evidence on an oral administration study, the median effective dose of sulforaphane (SFN) in mice is 175 µmol/kg/day (31 mg/kg/day), while human oral doses from broccoli sprout preparations range up to 847 µmol/day (2.14 mg/kg/day for a 70 kg adult), indicating biological activity and safety well below toxic thresholds. It was reported that the acute LD50 in mice via injection is 213 mg/kg (1203 µmol/kg), oral bioavailability is lower, making this a supplemental safety reference rather than a feed guideline [61]. In a study on the feed additive 3-nitrooxypropanol (3-NOP) given to beef cattle, researchers tested a range of increasing doses of the additive in the feed and observed how methane emissions changed with each dose. They used dose levels including 50, 75, 100, 150, and 200 mg 3-NOP per kg of feed dry matter, and found that the methane reduction effect increased with dose up to the highest levels tested [62]. As an illustrative example of formulation design, a hypothetical inclusion range of 50–100 mg SFN/kg feed was estimated from the available cross-species literature. This range should not be interpreted as a validated livestock recommendation but rather as a conceptual framework to guide future dose and response studies in target animal species. This is achievable using ionic gelation encapsulation, which typically achieves a loading efficiency of 95%, allowing the desired SFN dose to be incorporated into carriers that enhance stability and absorption [63]. Encapsulation ensures targeted release, bypassing ruminal degradation in ruminants, and protects SFN from pH > 5 and light-mediated degradation in non-ruminants before it reaches the stomach [64].

## 4. Measurable Efficacy in Animal Models

### 4.1. Quantifying Enhanced Bioavailability and Systemic Delivery

The primary objective is to enhance bioavailability. The reactive isothiocyanate bond in SFN renders it unstable, leading to rapid degradation in the digestive system and significantly hindering its distribution throughout the body [65]. Ionic gelation encapsulation effectively addresses this issue by creating a protective barrier. Chitosan–alginate systems designed for gastric resistance and intestinal release in feed models have demonstrated significantly improved recovery of intact SFN and its metabolites in plasma compared to the unencapsulated form [66]. The primary method to enhance the bioavailability of SFN, a crucial element in the efficacy of feed additives, is to optimize its release profile and safeguard it from degradation [67].

### 4.2. Modulation of Health and Resilience Markers

**Figure 6:**
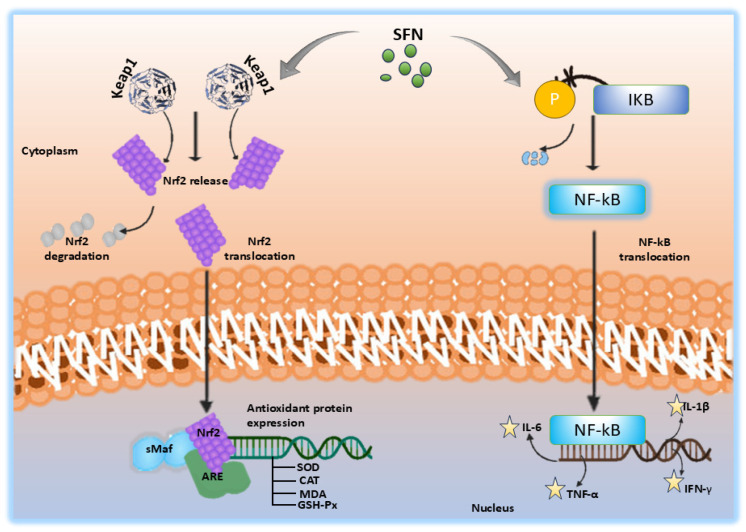
Mechanistic overview of sulforaphane’s bidirectional modulation of nrf2 and nf-κb pathways in cellular homeostasis. Sulforaphane change on the Nrf2/ARE pathway, which aids in the production of antioxidant proteins and phase II detoxification enzymes [71,72]. Concurrently, SFN stops the NF-κB pathway, which regulates anti-inflammatory gene expression and inhibits the progression of inflammation [73,74]. Arrows indicate activation, inhibition, or nuclear translocation events. SFN (sulforaphane) is shown modulating both Nrf2/ARE antioxidant signaling and NF-κB inflammatory signaling pathways. Color coding distinguishes cytoplasmic and nuclear compartments, while symbols represent key proteins, transcription factors, and cytokines involved in the signaling cascade.

Sulforaphane is a recognized stimulator of the Nrf2 (nuclear factor erythroid 2-related factor 2) signaling pathway in the biomedical literature (Figure 6). This route enhances the function of cellular antioxidant defense mechanisms and mitigates oxidative stress [68,69]. A recent study of goat mammary epithelial cells shown that SFN mitigated H_2_O_2_-induced oxidative stress and apoptosis by activating the AMPK and Nrf2 signaling pathways [70]. These mechanistic findings support the hypothesis that SFN may contribute to oxidative stress mitigation in animal tissues, although confirmation under practical livestock conditions remains limited.

**Figure 7:**
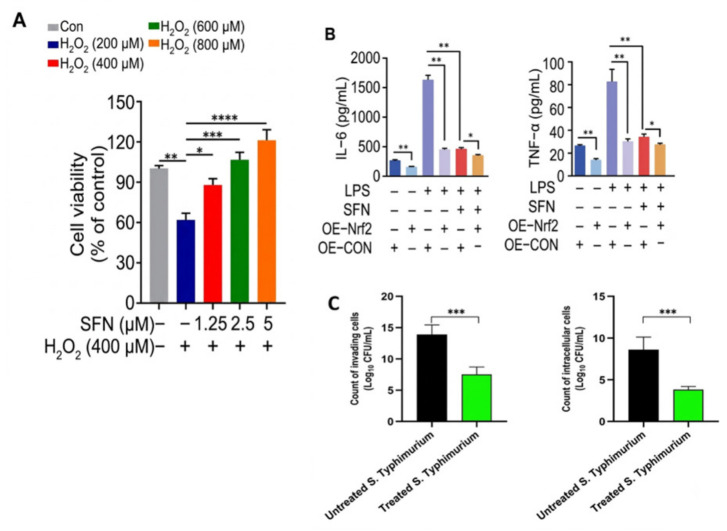
(**A**) Sulforaphane (SFN) enhanced the antioxidant defense system in primary goat mammary epithelial cells (GMECs). Reproduced from [70]. Licensed under CC BY 4.0. (**B**) SFN exerts anti-inflammatory effects through an Nrf2-dependent mechanism in LPS-stimulated HGFs. Bar colors represent different experimental groups as indicated in the figure legend (control, LPS, SFN-treated groups at increasing concentrations, Nrf2 overexpression (OE-Nrf2), and vector control (OE-CON)). Data represent IL-6 and TNF-α levels. Reproduced from [75] with permission from Elsevier. Copyright (2025). (**C**) SFN exerts antibacterial effects by targeting bacterial virulence rather than direct growth inhibition. Black bars represent untreated samples, while green bars represent Mycoplasma hyopneumoniae-treated conditions. SFN reduces pathogenicity without inducing selective pressure for antibiotic resistance. Reproduced from [76]. Licensed under CC BY- NC-ND 4.0. Statistical significance is indicated as follows: * *p* < 0.05, ** *p* < 0.01, *** *p* < 0.001, **** *p* < 0.0001.

In this effort, sulforaphane demonstrated a dose-dependent protective effect against oxidative stress. As shown in Figure 7A, treatment with SFN at concentrations of 1.25, 2.5, and 5 µM significantly mitigated hydrogen peroxide (H_2_O_2_)-induced cellular damage in HeLa cells, suggesting antioxidant activity under these in vitro conditions. Sulforaphane alone showed no toxicity. SFN + H_2_O_2_ groups showed significantly higher viability than H_2_O_2_ alone. Data are means ± SEM (n = 3), *p* < 0.05, *p* < 0.01 vs. H_2_O_2_ only [70]. In this process, ELISA quantification of IL-6 and TNF-α secretion in Nrf2-overexpressing HGFs treated with LPS and sulforaphane was performed, and the results are presented in Figure 7B as means ± SE of three independent experiments (*p* < 0.05, *p* < 0.01) [75]. This reveals that sulforaphane’s suppression of IL-6 and TNF-α is enhanced in Nrf2-overexpressing cells [76]. These findings indicate that SFN disrupts the bacterial Type III Secretion System (TTSS), which is essential for host cell invasion and intracellular survival. This anti-virulence strategy is particularly advantageous because it reduces pathogenicity without imposing selective pressure for antibiotic resistance, distinguishing SFN from conventional bactericidal agents (Figure 7C).

Besides its preventative qualities, increasing data suggests that SFN may directly influence growth and muscle development. In porcine satellite (muscle stem) cells, SFN treatment enhanced cellular proliferation, modified the expression of myogenic regulatory proteins, reduced histone deacetylase (HDAC) activity, and increased the acetylation of histones H3/H4. The researchers observed that SFN’s epigenetic effects may be advantageous for modulating muscle growth in swine [77]. This in vitro work indicates a molecular connection suggesting that SFN (or other compounds) may influence muscle development or tissue repair in animals, transcending simple protective roles.

In vivo, this yields measurable health advantages: a decrease in oxidative stress markers, an elevation in endogenous antioxidant enzymes, and a reduction in circulating pro-inflammatory cytokines [78,79]. A study conducted by Wang et al. (2020) demonstrated that hypoxic–ischemic (HI) injury in neonatal piglets results from impaired oxygen delivery and reduced cerebral blood flow, with oxidative stress acting as a major downstream contributor to neuronal damage [80]. In this research, post-insult administration of sulforaphane enhanced Nrf2-mediated antioxidant responses and increased neuronal survival, indicating its potential to mitigate oxidative-stress-related HI injury. Furthermore, SFN has proven efficacy in mitigating specific feed-related toxicities. In porcine intestinal cells, SFN alleviated deoxynivalenol (DON)-induced cytotoxicity by regulating spermine metabolism, decreasing oxidative stress, and lowering apoptosis, thus establishing a theoretical foundation for its use against mycotoxins [81,82].

### 4.3. Species-Specific Delivery Challenges and Outcomes

**Figure 8:**
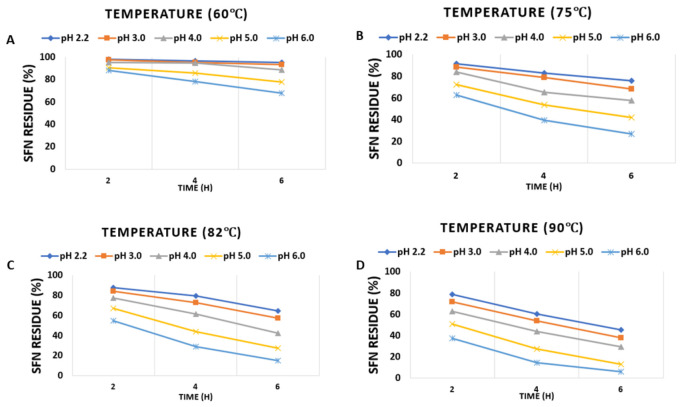
Sulforaphane retention over 6 h under various pH and temperature conditions. Data from Table 1 of were extracted and replotted as line graphs in panels (**A**–**D**) as indicated.

All animals metabolize SFN, but the intestinal milieu dictates its encapsulation [83]. Getting the maximum efficiency of sulforaphane—SFN—into the animal system has barriers—its inherent chemical instability during gastrointestinal transit and feed processing. Critically, SFN degradation follows a relationship with pH: SFN is stable under acidic conditions but degrades rapidly under a neutral to alkaline pH environment. This fundamental property dictates the degradation challenges across the different animal digestive systems [84]. Wu et al. [85] investigated the effect of pH on sulforaphane stability at different temperatures (see Table 1 in their study). For better clarity on these trends, the data obtained have been replotted as line graphs in Figure 8. As shown in Figure 8A–D, SFN was quite stable at low pH values and temperatures. At pH 2.2, even after heating at 60 °C for 6 h, more than 95.1% of SF was retained. However, retention decreased as pH and temperature increased. When the pH increased to 6.0, even at a temperature of 60 °C, 32.1% of SFN was lost within 6 h. After 6 h at 90 °C and pH 6.0, 6.0% of SFN remained (Figure 8D). Overall, increasing pH from 3.0 to 5.0 hastened SFN degradation, indicating that SFN is unstable at high temperatures, especially under high pH conditions [85]. In other words, SFN is more heat-stable in acidic food products.

A notable difference lies in the source of bioactivation. In non-ruminants, plant-derived myrosinase is typically inactivated in the stomach, and the production of SFN mostly relies on the hydrolysis of glucoraphanin by the gut microbiota [86].

In ruminants, the issue is exacerbated by the intricate microbial population of the rumen, which can rapidly decompose or alter unprotected compounds. To demonstrate efficacy, one must establish that it safeguards the rumen. Encapsulation techniques must effectively manage ruminal fermentation while disintegrating in the abomasum. This indicates that a universal release profile is ineffective; the system must be tailored to navigate or use the species-specific microbial ecosystem to ensure bioavailability beyond the rumen or intestines [87]. Recent strategies emphasize the employment of microbial-targeted delivery methods encapsulated in oxidized konjac glucomannan microspheres to directly enhance gut capabilities for making SFN, hence improving utilization rates [88].

Current evidence suggests that nano-encapsulated sulforaphane may support antioxidant responses, hepatic function, and physiological resilience under specific experimental conditions. In some studies, dietary SFN supplementation in pigs has been associated with improved growth-related parameters together with enhanced hepatic cellular responses, though these findings require more studies for confirmation and more species- specific data [89,90]. In broilers, related delivery systems have demonstrated beneficial physiological responses in challenge models; however, evidence linking these responses to improvements in productive performance remains limited [79]. These benefits are commonly associated with metabolic depletion caused by inflammation and oxidative stress. The effectiveness of these benefits is largely dependent on the stability of the encapsulated substance within the feed matrix during storage, as a product that degrades after manufacture would not ensure dependable field performance [91].

Taken together, the available evidence supports a formulation-level rationale for SFN encapsulation rather than a definitive prediction of field performance. Improved encapsulation efficiency, protection against degradation, and controlled release may increase the likelihood of SFN reaching relevant biological sites; however, these formulation advantages should not be interpreted as direct evidence of improved growth, productivity, feed efficiency, or disease resistance in livestock. At present, many reported effects are based on cellular systems, biomedical models, or limited animal studies. Therefore, livestock-related outcomes should be regarded as hypotheses requiring validation through controlled species-specific feeding trials that measure bioavailability, oxidative stress and inflammatory markers, safety, growth performance, and practical farm-level responses.

## 5. Considerations and Safety Regulatory

### 5.1. Regulations on Nanotechnology in Feed Additives

Nanotechnology is increasingly utilized in agricultural, animal feed, and food production. Examples include nano-encapsulated agrochemicals, antimicrobial coatings, and intelligent food packaging, all technologies relevant to delivering compounds like sulforaphane (SFN). Any future commercialization of nano-encapsulated SFN will depend heavily on navigating these regulatory frameworks. For SFN in particular, its nano-encapsulation for feed applications would fall under existing regulatory frameworks, but gaps remain. Various nations possess distinct regulations concerning nanotechnology. The European Union (EU), together with Switzerland, is among the few jurisdictions with nano-specific provisions, regulations on food, feed, and chemicals have been amended to incorporate nanotechnology. Conversely, non-EU countries such as the U.S. often govern nanomaterials within the framework of broader chemical and food safety regulations. They frequently depend on industry directives and voluntary adherence [92,93,94,95]. In Table 1, it gives the necessary information pertaining to the guidelines of nanotechnology in feed additives including implications for SFN-based products.

The Food and Drug Administration (USFDA) regulates the majority of regulations concerning nanotechnology in food and agriculture in the United States. The FDA evaluates nano-enabled products such as SFN nano-formulations according to existing rules, such as the Federal Food, Drug, and Cosmetic Act, despite the absence of specific nano legislation in the U.S. It has advised makers to communicate promptly when nanoscale alterations may impact safety or efficacy [96]. These are not legally enforceable, and their enforcement largely relies on conventional risk-based evaluations which may not fully capture the unique properties of nano-encapsulated SFN. The Environmental Protection Agency (EPA) is responsible for ensuring the safety of nanomaterials, particularly when utilized in pesticides or released into the environment. Despite ongoing study, U.S. regulatory authorities continue to encounter issues with incomplete data and the absence of standardized testing methodologies for nanoscale substances [97].

The European Food Safety Authority (EFSA) operates within a more structured and meticulous framework. The EFSA is responsible for conducting nano-specific risk evaluations prior to the sale of products inside the EU. This is particularly applicable to novel foods, feed additives, biocides, and substances that interact with food of relevant to nano-encapsulated SFN as a feed additive [98]. For SFN specifically, this means any commercial nano-formulation would require pre-market approval through EFSA’s nano-specific assessment process [99]. Experts assert that despite improvements, it remains essential to enhance capacity, standardize practices across nations, and engage the public to effectively address the risks and ethical concerns associated with nanotechnology in food systems, challenges that apply directly to the future adoption of nano-encapsulated SFN in animal nutrition [100,101].

### 5.2. Major Challenges for Approval of Nanotechnology in Animal Feed

Numerous scientific, regulatory, and methodological challenges must be addressed prior to the application of nanoparticles in animal feed. A significant issue is the lack of standardized, nano-specific testing methodologies; traditional toxicological methods often fail to consider the unique properties of nanoparticles, such as altered bioavailability, surface reactivity, and the potential for bioaccumulation in animal tissues. Regulatory bodies like the EFSA require data on particle size, toxicokinetic, genotoxicity, and chronic oral toxicity; however, established approaches for producing this data are often inadequate [92,102]. Compounding the issue, numerous nano-enabled feed applications remain in the experimental or research phase, indicating a lack of sufficient long-term safety data for both animals and humans consuming the products [93].

A significant issue is the variation of regulations throughout different regions of the world, coupled with insufficient coordination among nations. The EU has incorporated nano-specific controls into its feed legislation, whereas most non-EU countries, notably the U.S., govern nanoparticles under general food and chemical safety laws that do not explicitly address nanoscale characteristics [94,98]. This fragmented approach complicates international trade and creates ambiguity for developers. In the United States, the FDA has issued certain rules regarding nanoparticles; however, a regulatory framework for nanotechnology in feed is absent, and there is insufficient collaboration among regulators, industry, and academics [95]. The absence of centralized databases, labeling rules, and defined post-market monitoring methods complicates transparency and risk management. Consequently, regulatory ambiguity remains a significant obstacle to the approval and safe utilization of nano-enabled feed items, despite their increasing innovation.

### 5.3. Toxicity and Safety

Nanotechnology has attracted increasing attention in agriculture and animal feed due to its potential applications in improving nutrient delivery, enhancing bioavailability, and supporting animal health. Engineered nanomaterials (ENMs) have been reported to improve feed efficiency and biological absorption through their unique physicochemical properties. However, their small size and high surface area confer increased chemical reactivity, which may also raise safety concerns. Multiple studies have demonstrated that nanoparticles can enter biological systems through ingestion, inhalation, and dermal exposure pathways, and may subsequently translocate to organs such as the liver, spleen, and lungs. Once internalized, nanoparticles can interact with cellular components and induce oxidative stress, inflammatory responses, and tissue damage through reactive oxygen species (ROS)-mediated mechanisms. These toxicological effects are widely discussed in nanotoxicology literature, emphasizing the need for careful safety evaluation of nano-enabled feed applications [103,104]. The ability of nanoparticles to penetrate biological membranes and infiltrate cells increases the risk of genotoxic and metabolic disruptions [105]. Research indicates that nanoparticles might adversely affect reproductive health and the development of progeny in animals, highlighting another aspect of reproductive toxicity [106]. Indirect exposure to animal products presents a considerable risk to human health, especially due to the inadequate understanding of the breakdown or accumulation of nanomaterials in the food chain [107]. Moreover, whereas acute toxicity is frequently negligible, prolonged exposure, particularly via inhalation, has been demonstrated to adversely affect the lungs and other bodily systems [108]. A summary of regulatory criteria for nanomaterial approval in animal feed is presented in Table 2. Moreover, whereas acute toxicity is frequently negligible, prolonged exposure, particularly via inhalation, has been demonstrated to adversely affect the lungs and other bodily systems [109]. This complicates the determination of safe exposure levels for both humans and animals.

Innovative approaches to ensure the safety and legality of nanotechnology in animal feed involve a combination of regulatory frameworks, risk assessments, and practices that foster sustained innovation. An increasing body of research emphasizes the necessity for explicit regulations to govern the growing application of nanomaterials in feed; due to the potential health and environmental risks they present. Regulatory frameworks are evolving globally. Prior to the sale of a product, firms must demonstrate the safety of nano-based feed additives through systematic risk assessments. This review includes the assessment of nanoparticle bioavailability, accumulation, and possible toxicity in both cattle and humans consuming animal products [94]. It is recommended that industrial feed manufacturers implement frameworks such as Good Manufacturing Practice (GMP) and Hazard Analysis and Critical Control Points (HACCP) to maintain elevated safety standards and monitor contamination risks [117]. Moreover, research has supported the integration of a “Safe-and-Sustainable-by-Design” approach in nanomaterial manufacturing. This entails examining safety and environmental impacts from the inception of the design process to ensure the product’s durability and regulatory compliance [118]. Researchers have emphasized the necessity for targeted investigations into the effects of nano-feed additives on specific animal species, as the existing data are insufficient for thorough regulatory assessments [119]. These strategies collaborate to ensure that emerging technologies comply with health, environmental, and legal standards.

## 6. Future Direction and Opportunities

While previous research on sulforaphane (SFN) has focused heavily on biomedical and cancer-related applications, there is a significant gap in understanding its potential for livestock feed and animal health [10]. Prospects and trajectories for the future advancements in the delivery of sulforaphane hinge on the development of more stable and efficient nanocarrier systems capable of overcoming SFN’s inadequate solubility, instability, and rapid degradation [8]. Nanocarriers developed in pharmaceutical contexts, such as micellar mPEG–PCL and solid lipid nanoparticles, illustrate the potential for modulating SFN release kinetics. Similar strategies could be adapted for livestock feed to enhance bioavailability and controlled release, though their use in animal nutrition remains unexplored [120,121,122].

Current research necessitates the enhancement of encapsulation efficiency, release profiles, and targeting specificity of nanocarriers via advanced formulations such as dual-drug systems or polymer–lipid hybrids [123]. Innovations like prolamin-based composites [124] and microencapsulation with gelatin–pectin complexes [25] show promise in food-grade delivery applications. Encapsulation within hydrogels [125] or coating with basil seed gum [28] further improve mucosal adhesion and controlled intestinal release. Future research should also prioritize in vivo validation and toxicological profiling in livestock models, bridging the gap between bench research and practical applications in animal production. The use of plant-derived vesicles [126] as both carriers and bioactive agents opens novel avenues for natural and multifunctional delivery systems.

Development of robust, food-grade nanocarriers for SFN can improve its incorporation into feed, ensuring consistent exposure and protection from degradation during processing and digestion. Additionally, systematic in vivo studies are needed to assess growth performance, immune function, and resilience under practical farm conditions. Expanding the evidence base in diverse livestock species will address the current knowledge gap and support regulatory approval and adoption of SFN-enriched feed additives. Overall, aligning future perspectives with animal feed and health will bridge the translational gap from biomedical studies to practical, safe, and effective applications in livestock systems.

## 7. Conclusions

Nanotechnology-driven encapsulation methods for sulforaphane (SFN) highlight numerous significant discoveries and their potential implications for animal health and may contribute to sustainability though evidence remains limited. A notable outcome is that the preliminary potential of nano-encapsulated SFN as a functional feed addition may contribute to improved animal health under experimental conditions. Nanocarriers such as chitosan with alginate give potential indications for enhancing the stability, bioavailability, and controlled release of SFN within the gastrointestinal tract. This advancement allows SFN to execute its antioxidant and anti-inflammatory roles, improving immune responses, nutrient absorption, and resistance to oxidative stress and toxins, such as mycotoxins, while some porcine studies have associated dietary SFN supplementation with improved growth parameters and upregulated hepatic cellular responses; these preliminary findings require substantiation through additional research and species-specific evidence for animal feed.

Despite good results more studies are needed to fully comprehend the efficiency, release kinetics, and targeting specificity of SFN-loaded nanocarriers for different animal species. There is an urgent necessity for in vivo validation of these technologies and the establishment of regulations to ensure their safety, utility, and minimal environmental impact. The research suggests that further exploration of plant-based nanocarriers and enhanced formulations could produce safer and more effective feed additives, thereby advancing animal welfare and sustainability in livestock agriculture.

## Figures and Tables

**Table 1:** Studies of encapsulation efficiency of sulforaphane and related isothiocyanates.

Active 17.	Synthesis Method	Representative Carrier	Encapsulation Efficiency (%)	Reference
Sulforaphane	Complex coacervation	Gelatin/pectin	17.9 ± 1.3	[25,48]
Benzyl isothiocyanate	Emulsion ionic gelation	Chitosan nanoparticles	64.68 ± 4.7	[49,50]
Sulforaphane	Spray-drying microencapsulation	Gum Arabic-based wall system	39.1 ± 2.6	[27,51]
Allyl isothiocyanate	Emulsification	Calcium alginate beads	82.8	[52,53]
Sulforaphane	Polymeric micelles	PCL–PEG–PCL	87.1	[54,55]
Sulforaphane	Polymeric nano-delivery	mPEG–PCL	86.0 ± 1.6	[54,56]

**Table 2:** Regulatory criteria for nanomaterial approval in animal feed.

Parameter	Why It Matters	Regulatory Enforcement/Guidance	Reference
Particle size distribution	Determines whether material qualifies as a nanomaterial (<100 nm for 50%+ particles)	EU definition: ≥50% of particles by number must be 1–100 nm to qualify as a nanomaterial	[110,111]
Surface area (BET method)	High surface area influences reactivity and bioavailability	Required for nanomaterial identification in EFSA and OECD guidelines	[112]
Agglomeration and aggregation state	Affects particle behavior in biological systems and toxicity	EFSA requires evaluation in relevant media (e.g., feed matrix or digestive fluids)	[113]
Solubility and dissolution rate	Determines whether particles persist at nanoscale in the GI tract	Essential to decide whether nano-specific risk assessment is needed	[114]
Shape and aspect ratio	Rods, tubes, and fibers may behave differently than spheres	Required in OECD testing guidance and EFSA assessment	[115]
Surface charge (zeta potential)	Affects interaction with cells and proteins	Recommended by EFSA for risk and biointeraction studies	[116]
Impurities and chemical composition	Trace contaminants can influence toxicity and regulatory acceptance	EFSA and OECD require full elemental/chemical profiling	[115]
Bioavailability	Critical for feed effectiveness and systemic exposure	Often evaluated through in vitro or in vivo digestion models	[102]
Stability in biological media	Affects reliability of exposure and toxicity predictions	Testing in feed and GI-like conditions is recommended	[113]
Genotoxicity	Required for safety clearance of nanomaterials	EFSA: in vitro genotoxicity, 90-day rodent toxicity, toxicokinetic	[102,114]

## Data Availability

No data was used for the research described in the article.
